# How does ‘autistic burnout’ feel? A qualitative study exploring experiences of earlier and later-diagnosed autistic adults

**DOI:** 10.1177/13623613261422117

**Published:** 2026-02-28

**Authors:** Dorota Ali, Will Mandy, Francesca Happé

**Affiliations:** 1 King’s College London, UK; 2 University College London, UK

**Keywords:** autism, autistic burnout, burnout, fatigue, mental health, reflexive thematic analysis

## Abstract

‘Autistic burnout’ is increasingly conceptualised as an experience of severe exhaustion, increased sensory difficulties and need for solitude, resulting from not having needs met across contexts. We interviewed 20 autistic adults (eight diagnosed with autism in childhood) about their experiences of autistic burnout. In this reflexive thematic analysis, we focused on how burnout felt, comparing the experiences of those diagnosed in childhood versus adulthood. We created five themes. We understood burnout to be, at times, (1) *a powering down and/or* (2) *the overactivation, of the mind and body*, resulting in (3) *a craving for sensory and social rest*. For those diagnosed in adulthood, burnout seemed to be experienced as more chronic and confusing. Some participants diagnosed in adulthood (4) *made the world more bearable by using substances*, coping with the contributors to and effects of burnout. Especially affecting those diagnosed in adulthood, (5) *not knowing why this was happening took a (sometimes dangerous)* toll; for a few, it led to contemplating suicide. This study brings new insights into burnout experiences of adults diagnosed with autism at different life points.

**Lay abstract:**

Some autistic people experience severe exhaustion as a result of not having their needs met that sometimes prevents them from being able to take part in daily life. Some people refer to this as ‘autistic burnout’. In this study, we spoke to 20 autistic adults, eight of whom were diagnosed with autism in childhood. We analysed our participants’ interviews through an approach called reflexive thematic analysis. Through this process, we created five themes around the question of how autistic burnout felt for these participants. We were also interested in how participants diagnosed with autism in childhood versus in adulthood described their burnout experiences. This is because research has shown that not having an autism diagnosis earlier in life could, indirectly, lead to not understanding one’s own needs accurately and not having the right support from others. The five themes we created were (1) the powering down of the mind and body, (2) the overactivation of the mind and body, (3) craving social and sensory rest, (4) making the world more manageable by using substances and (5) not knowing why this is happening to you can take a (sometimes dangerous) toll. Themes 1, 2 and 3 seemed to be shared between participants regardless of age at autism diagnosis. However, these experiences seemed to be more disabling for participants diagnosed in adulthood. Themes 4 and 5 related particularly to those diagnosed with autism in adulthood. This study adds an important insight: perspectives on burnout experiences from adults diagnosed with autism at different life points.

## Introduction

Using a community-based participatory approach, Dr Dora Raymaker and colleagues introduced ‘autistic burnout’ to the research community. Raymaker et al. (2020) conceptualised autistic burnout as a chronic condition consisting of severe exhaustion, loss of skills and increased sensory sensitivity. Since the publication of Raymaker et al.’s (2020) qualitative study, others have worked to define (e.g., Higgins et al., 2021), understand the aetiology of (e.g., Mantzalas, Richdale, & Dissanayake, 2022) and create psychometrically sound ways to measure autistic burnout (e.g., Mantzalas et al., 2024).

Albeit with differing emphases, most studies to date have defined autistic burnout in terms of physical and mental exhaustion, loss of abilities, worsened sensory difficulties and a need to withdraw socially (Arnold et al., 2023a, 2023b; Clarey et al., 2025; Higgins et al., 2021; Mantzalas, Richdale, Adikari, et al., 2022). Some have highlighted its distinction from depression, suggesting anhedonia and severe exhaustion as differentiating factors, with the former more prevalent in depression, and the latter in autistic burnout (Raymaker et al., 2020). Autistic burnout negatively impacts mental and physical wellbeing, with some autistic people contemplating suicide (Arnold et al., 2023b; Higgins et al., 2021).

Through primarily qualitative studies, autistic burnout has been increasingly understood to be linked to various stressors, such as camouflaging. Camouflaging – used here as an umbrella term for various strategies that result in the suppression of intuitive ways of being and the taking on of less intuitive but more socially approved ones (Ai et al., 2022) – can be very draining for autistic people. Some autistic people begin camouflaging in childhood (Ross et al., 2023), as a result of discrimination due to being perceived as different, potentially impacting their self-concept (Chapman et al., 2022; Howe et al., 2023). Indeed, some autistic children have been described to experience high fatigue levels after sensory and social exposure (Keville et al., 2021; Phung et al., 2021).

Synthesising some of the autistic burnout studies, Mantzalas, Richdale and Dissanayake (2022a) generated an autistic burnout risk and protective factors model, suggesting that some variables could be both a risk and a protective factor, dependent on circumstances. For example, camouflaging could lead to the securing of employment, which could lead to financial stability. However, too much camouflaging without other supportive factors could contribute to burnout, which could lead to loss of employment and financial instability. Mahony and O’Ryan (2022) further theorised camouflaging as one form of early life stress that leads to high ‘allostatic load’ (the mounting impacts of long-lasting stress on health) for autistic people. The resultant autistic burnout linked to this allostatic overload was proposed to then increase the risk of psychopathology, such as depression, as well as suicidality (Mahony & O’Ryan, 2022). These findings and theories are pertinent, as autistic people across the lifespan are at higher risk of experiencing both physical (Cashin et al., 2016) and mental health conditions (Lai et al., 2019), as well as adverse experiences, such as peer violence, parental mental health difficulties (Hartley et al., 2023) and suicide mortality (Brown et al., 2024), the latter especially when co-occurring depression is present (Kim et al., 2024).

Conceptually, autistic burnout has also been increasingly differentiated from occupational burnout, a state with which it shares its name – and the original burnout construct. Although a frequently explored experience, occupational burnout has occupied an uncertain position within research and clinical practice, being neither a universally agreed construct (Guseva Canu et al., 2021) nor, in the United Kingdom, a medically recognised condition (Bianchi et al., 2015). The two experiences share some similarities (primarily, exhaustion), yet autistic burnout is proposed to be a more global experience, not associated with one context (work) – arguably akin to Bianchi et al.’s (2014, p. 359) ‘multi-contextual syndrome’ of burnout – particular to autistic people due to their experience of the world and the world’s treatment of them.

Furthermore, in the United Kingdom within the past 20 years, there has been increased recognition and diagnosis of autistic people in adulthood (Russell et al., 2022). Not being identified as autistic until adulthood can occur for multiple reasons. Girls and women have been historically under-diagnosed due to diagnostic tools and criteria being created with mostly boys and men in mind, with autistic girls sometimes presenting differently to boys (Cook et al., 2024). Other factors that may delay diagnosis include presenting with subtler socio-communication difficulties in childhood (including, potentially, through camouflaging these difficulties; Dworzynski et al., 2012), lower socio-economic status (Avlund et al., 2021), belonging to an ethnic minority (Malwane et al., 2022), as well as geographical location, policies and service availability (Daniels & Mandell, 2014).

Several quantitative studies have explored the association between age at autism diagnosis (and/or age at learning one is autistic) and various wellbeing, health and life outcomes. So far, the results have been mixed. For example, Oredipe et al. (2022) found that earlier knowledge that they were autistic was associated with higher self-reported wellbeing and quality of life in a convenience sample of 78 autistic students. Likewise, Diemer et al. (2025) found that autistic women diagnosed in adulthood were more likely to experience eating disorders, depression, anxiety and substance use than autistic women diagnosed in childhood (in a sample of 1,424 women, a third of whom were diagnosed with autism in childhood). Yet these findings have not been consistent: in a replication of Oredipe et al.’s (2022) work, Leung et al. (2023) did not find the age at which their 300 autistic participants learned they were autistic to significantly and uniquely predict wellbeing and quality of life. Looking at recent qualitative work, adults diagnosed later in life have, however, consistently expressed a feeling of regret about not learning that they were autistic sooner, as it could have helped them know themselves better, understand the challenges they faced and potentially provide access to more appropriate support (Nayyar et al., 2025).

Although qualitative studies do not usually aim at generalisability (Drisko, 2024), it is nevertheless important to sample a range of voices. Currently, those diagnosed with autism in childhood appear to be under-represented in autistic burnout research (Ali et al., 2025). Given the increasing qualitative evidence that late-diagnosed autistic people feel regret for not having an earlier diagnosis, as it impacted negatively on their wellbeing, and some studies suggesting fewer mental health conditions in those diagnosed with autism in childhood versus adulthood, it is important to extend this exploration to research on autistic burnout. Our study sought perspectives from both earlier and later-diagnosed autistic adults across the age spectrum, who self-identified with an experience of autistic burnout. We explored potential differences in autistic burnout associated with autism diagnosis timing, given the possibility that, since these two groups may differ on developmental experiences (e.g., more/less appropriate support in childhood), their burnout occurrences or trajectories may also differ (Stagg & Belcher, 2019). Our aim was to conduct reflexive thematic analysis (RTA) on interview data collected from autistic adults, guided by the following research question:

**Research Question (RQ):** How does ‘autistic burnout’ feel in the mind and body, and do these experiences differ between autistic participants diagnosed in childhood versus adulthood?

## Method

### Participants

Twenty participants took part in semi-structured interviews. Eight participants were diagnosed with an autism spectrum condition (ASC) in childhood (mean age at diagnosis = 10 years (4.18), range = 4–16 years), while 12 were diagnosed with ASC in adulthood at age 18 or over (mean age at diagnosis = 42 years (12.24), range = 28–66 years). Generally, we had a balance of genders between groups, although the childhood-diagnosed participants were, on the whole, younger and identified more frequently with a minoritised ethnicity. Participants also reported several medical conditions, with the most frequently reported condition for both childhood-diagnosed participants (37.5%) and those diagnosed in adulthood (50.0%) being attention deficit hyperactivity disorder (ADHD). Only two participants reported not having a diagnosis of a medical condition. Table 1 shows the full demographics by participant. Note that participants were assigned pseudonyms, and their current age and age at autism diagnosis are reported in age bands to protect their anonymity; to further protect privacy, in section ‘Findings’, we do not always connect a quote to a specific participant. Participants diagnosed with autism in childhood are indicated with a ‘_ch_’, while those diagnosed in adulthood are indicated with an ‘_ad_’.

**Table 1.** Demographic information of the participants.

Participant pseudonym	Age	Gender	Ethnicity	Highest education level	Age at autism diagnosis	Number of burnout episodes
*Andrew_ch_*	35-44	Cis Male	Caucasian	Secondary education	11-15	Multiple
*Ashley_ch_*	35-44	Non-binary	White British/Other	Master’s-level degree	6-10	Multiple
*Dan_ch_*	18-24	Cis Male	White	Undergraduate degree	6-10	Multiple, short
*Ella_ch_*	18-24	Cis Female	White British	Secondary education	6-10	Multiple, short
*Esther_ch_*	18-24	Cis Female	Not reported	Secondary education	11-15	Multiple, short
*Lee_ch_*	35-44	Cis Male	Asian	Master’s-level degree	6-10	Multiple
*Molly_ch_*	25-34	Cis Female	White and Asian	Undergraduate degree	16-20	Multiple
*Oliver_ch_*	18-24	Cis Male	Chinese	Undergraduate degree	0-5	Multiple, short
*Alex_ad_*	35-44	Other	White British	Master’s-level degree	36-40	Multiple
*Diana_ad_*	25-34	Cis Female	White	Master’s-level degree	26-30	Two
*Helen_ad_*	55-64	Cis Female	White British	Undergraduate degree	56-60	Multiple
*Henry_ad_*	55-64	Cis Male	White English	Undergraduate degree	56-60	Multiple
*Lily_ad_*	25-34	Cis Female	White British	Undergraduate degree	26-30	Two
*Luke_ad_*	45-54	Cis Male	White British	Undergraduate degree	36-40	Multiple
*Max_ad_*	45-54	Cis Male	White British	Undergraduate degree	46-50	Multiple
*Nathan_ad_*	35-44	Cis Male	White British	Secondary education	31-35	Multiple
*Poppy_ad_*	65-74	Cis Female	White British	Master’s-level degree	66-70	Unclear
*Robert_ad_*	45-54	Cis Male	White Welsh	Undergraduate degree	41-45	Multiple
*Samuel_ad_*	45-54	Cis Male	White British	Undergraduate degree	41-45	Multiple
*Zoe_ad_*	35-44	Cis Female	White British	Undergraduate degree	26-30	Multiple

### Recruitment and data collection

The study received ethical approval from the Health Faculties Research Ethics Subcommittee at King’s College London. Participants were recruited through an advert shared with the Autistica Network, a network consisting of autistic people that have opted in to receive research recruitment advertisements. The inclusion criteria were (1) being 18 years or over, (2) having a self-reported clinical diagnosis of an autism spectrum condition, (3) self-identifying with an episode of autistic burnout, (4) residing in the United Kingdom, (5) having safe Internet access and (6) being able to take part in English. Participants were asked to contact D.A. if they wanted to receive the study information sheet and to express interest.

Initially, we selected participants on a first-served basis. Once we recruited half of our proposed sample, we decided to take a more purposive sampling approach, contacting prospective participants (who had filled out a form) in such a way as to try to balance the group by age, age at autism diagnosis, gender and ethnicity. This was in order to include a diverse sample, capturing varied experiences from across the spectrum. Participants read an information sheet which was e-mailed to them, gave digital online consent via e-mail and filled in an online demographics questionnaire hosted on Qualtrics. They were also asked if they wished to be recontacted to give feedback on the initial analysis. We did not provide a definition of ‘autistic burnout’ to our participants during recruitment (such as in the information sheet), as we wanted to understand how the participants described autistic burnout unprompted.

The semi-structured interviews took place online via Microsoft Teams between February and March 2024. In total, 19 participants took part in the interviews via video-calling and through spoken language, while one participant chose to take part via video-calling with their camera switched off and typing their responses. The interview schedule (available in the Supplementary Materials) was structured into before, during and after autistic burnout, with questions probing participants about their felt experiences and retrospective reflections. The interviews were conducted and recorded by the first author and automatically digitally transcribed in Microsoft Teams. The first author checked and corrected the transcripts. The recordings were subsequently deleted, and all identifying information was removed from the transcripts. The participants were sent a £10 gift voucher in appreciation of their time.

### Positionality and data analysis

RTA, as developed by Braun and Clarke (2022), was the chosen qualitative methodology for this study. The analysis was conducted by the first author, with others providing feedback throughout. RTA is utilised by researchers to create patterns within given qualitative data, telling a meaningful, coherent and justifiable story (Braun & Clarke, 2022). An important aspect of RTA is that the researcher(s) bring their own understandings and interpretations to the data, a necessary part of the process (Braun & Clarke, 2022).

Our theoretical position was that of critical realism (Bhaskar, 2008; Walker, 2017). Although we understand reality as existing outside of subjective observations and interpretations, we also believe our observations and interpretations mediate that reality. Furthermore, we broadly understand autism through a ‘neurodiversity’ lens, which has influenced this analysis. Rather than our starting point of understanding being that sensory and socio-communication differences are an inherent pathology, we assume there is a diversity of minds and bodies in the world, with no one body-mind being more ‘normal’ (Pellicano & den Houting, 2022). Nevertheless, we do not reject the suffering that some experiences and conditions bring outside of social barriers, and we continue to reflect on and revise our understanding of the contradictions and complexities that are raised by ‘illness’, ‘health’ and ‘disability’ (e.g., Clare, 2017).

We were also a neurodiverse team, with the first author bringing both academic knowledge and lived experience of neurodivergence and burnout-relevant episodes. Throughout the process of this study, the first author engaged with a reflexivity journal, memos and discussions with the rest of the team, as well as wider reading. This helped to critically reflect on inherent (and changing) assumptions throughout the analysis process. For example, their experiences in terms of gender, ethnicity, socio-economic status and own specific experiences with neurodivergence and how they related to the participants’ accounts were recursively examined.

The first author coded the data through several rounds of immersion with the transcripts. Through this initial process, we decided that, rather than focusing on autistic burnout and gender as a point of comparison (e.g., do men and women describe autistic burnout differently?), which was the initial aim, we would primarily explore differences between earlier diagnosed participants and later-diagnosed participants. Through data familiarisation and coding, this difference seemed more relevant in the data collected. We took both an inductive and deductive approach to the analysis. Through phases, the first author looked at data trying to closely align with participants’ descriptions and experiences, while, at times, taking into account emerging concepts linked to autistic burnout (e.g., ‘camouflaging’), using them as tools to see if the data could be understood further through their lens. All data was analysed together, though, at several points throughout the analysis process, data from those diagnosed with autism in childhood and those diagnosed in adulthood was explored separately to see if we interpreted any meaningful differences between the two groups (Braun & Clarke, 2022, p. 127).

### Community involvement

The initial interview schedule was shared informally with two autistic colleagues who provided feedback, while two other autistic people, contacted through the lab group’s network, also shared their thoughts. The latter were re-imbursed for their time. One of our consultants was a cisgender Pakistani female in her late 20s diagnosed with autism in her mid-20s; no demographic information is available for the second consultant. From the four consultations, we made several changes to the interview schedule. For example, we created a short working definition of ‘autistic burnout’ to give to a participant in case they needed a prompt:

Autistic burnout has been described as extreme mental and physical exhaustion that occurs as a result of ongoing life stress that is not resolved. Some autistic people describe that some of their difficulties get worse (for example, sensory sensitivity); some autistic people describe needing to have more time to themselves, away from social situations.

Participants were also asked if they wished to be recontacted to provide feedback on the initial themes. The feedback questions asked were about the benefits of the findings for the autistic community, the benefits of the findings for non-autistic people, the perceived appropriateness of language use and any other negative or positive feedback about the themes. Sixteen of the original 20 participants responded and were re-imbursed with a £10 gift voucher. The key point from the feedback was that we should communicate the difference between chronic autistic burnout and briefer burnouts more strongly.

## Findings

**Figure 1.**
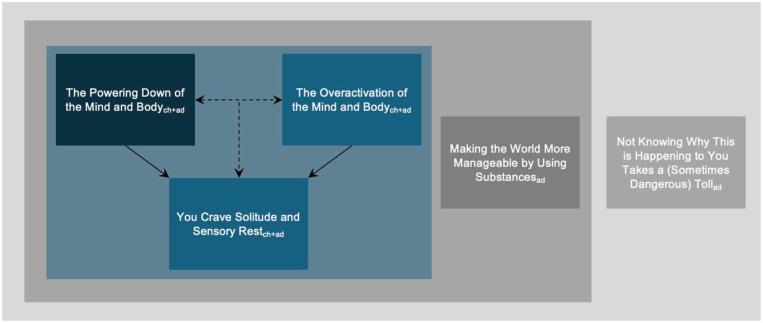
Conceptual thematic map of how we understood autistic burnout to feel in the body and mind of the participants. Please note themes relevant to both the childhood- and adulthood-diagnosed groups are indicated with a ‘ch + ad’, while themes relevant primarily to the adult-diagnosed group are indicated with an ‘ad’.

Figure 1 shows a conceptual thematic map showing our five generated themes. Many of our participants reported experiencing other aspects of neurodivergence as well as illness, health conditions and major physical and mental transitions. Although we did not generate a coherent theme from these experiences, we note these for context, as we perceived them to be woven into the autistic burnout experience.

### Theme 1. The powering down of the mind and body

We interpreted the powering down of the mind and body as one of the key experiences of autistic burnout, which involved feeling exhausted, emptied of energy and a slowing down:

*‘Zapped’ (Andrew_ch_), ‘extremely tired all the time’ (Samuel_ad_), ‘feeling exhausted, very fragile, and worn out; physically and mentally absolutely flattened’ (Henry_ad_), ‘no energy, [. . .] empty, [. . .] nothing left’ (Lily_ad_) and ‘truly fried*’ (Dan_ch_) were some of the ways participants expressed their energy loss and consequent exhaustion. For some, daily activities ‘*were much more difficult to do*’ (Nathan_ad_), as their energy depleted faster than usual, without sufficient replenishment. Poppy_ad_ noted:

*‘It’s, more or less, like, your muscles just are not charging up. [. . .] My battery is totally drained, and I can’t recharge it. That’s what it feels like. [. . .] Everything is an enormous Everest effort*’.

Participants diagnosed in childhood were more likely to relate to this energy loss and exhaustion as abrupt and short-lasting. Sometimes, these participants expressed their experience of autistic burnout as overlapping, or synonymous with, ‘meltdowns’, experiences of acute overstimulation, where control and abilities can be lost, and exhaustion can follow. Lee_ch_ explained:

‘*I’m not gonna lie, ‘cause it is quite difficult to separate between the two. [. . .] I guess, burnout, it’s a bit, like, you just have no energy left afterwards*’.

Many participants diagnosed in adulthood referred to some of their burnouts as lasting months or years, rather than days or weeks, as was the case for some of those diagnosed in childhood. It is important to note, however, that participants diagnosed in childhood were overall younger than those diagnosed in adulthood, which may have created a difference of experience due to age.

Tied to this felt depletion of energy and resultant exhaustion was a wide-ranging loss of usual abilities, which seemed to affect participants regardless of age at diagnosis. This included abilities relating to processing information (‘*Complex information that I could take a moment and be able to work out, now, I just can’t*’. – Samuel_ad_), planning the day (‘*[. . .] in the morning, I’ll, sort of, script through the day. But, you know, even doing that becomes too much*’. – Max_ad_), taking part in self-sustaining activities (‘*I would stop personal hygiene. Stopped being able to eat*’. – Helen_ad_), making decisions (‘*I wouldn’t be able to decide what I need [from the shop]*’. – Diana_ad_) and being able to process and produce speech (‘*[. . .] verbal communication gets very, very difficult [. . .]’* – Dan_ch_). Trying to assimilate to communities around them, and mask both their burnout and their autistic differences, was an experience expressed by several participants. This ability too was lost during burnout, which could leave a participant feeling vulnerable and exposed. We interpreted this loss of abilities as being connected within the same process, which involved shutting down of ‘*executive functioning*’ (Esther_ch_). Ashley_ch_ described:

*‘I’m having difficulties to control my speech, my emotions. I have difficulties to control my repetitive behaviour. I have difficulties to control pretty much everything*’.

### Theme 2. The overactivation of the mind and body

Another experience we interpreted as important to autistic burnout was the overactivation of the mind and body, which was again described by participants regardless of whether they were diagnosed with autism in childhood or adulthood. For some participants, it seemed as if the depletion of energy resulted in the breaking down of mechanisms that made external and internal stimuli more manageable. We interpreted this to result in further overwhelm and exhaustion, deepening autistic burnout.

A sense of restlessness, tension and overwhelming anxiety seemed to permeate across some participants’ experiences during the built-up to and the experience of burnout. Samuel_ad_ described feeling ‘*really hyper [. . .] everything’s tense [. . .] I get very, very hot*’, while Ella_ch_ reflected, ‘*[. . .I] fidget a lot, like, all the time. Getting very restless as well. [. . .] it just feels so weird just to relax*’. Luke_ad_ too found it ‘*more and more difficult to switch off properly*’. Inability to turn the mind and body off also affected some of the participants’ sleep, intensifying exhaustion. Andrew_ch_ shared his experience:

*‘I actually tape over lights as well, on computers, and things. [. . .] And I found that wasn’t still working. [. . .] What that then means is that my quality of sleep is not great, so I will not get refreshment from sleep. [. . .] [thoughts are] still running through my head*’.

Together with the felt decrease in control of one’s mind and body was an increase in pain associated with social and sensory input. Across participants, there was a sense that a ‘*filter, in general, [was] off*’ (Ashley_ch_), leading to ‘*more sensory issues*’ (Zoe_ad_), where ‘*bright lights are brighter, hot rooms are hotter, [. . .] clothes are more uncomfortable, sudden noises are louder*’ (Samuel_ad_). Dan_ch_ shared how, during burnout, he experienced the minutest of unforeseeable sounds:

*‘I find more difficulties in, like, processing noise. [. . .] Because any sort of simple, just pin drop, sometimes, is like, uhh, what was that?’*

This sensory pain could increasingly be followed, for some, by agitation and anger, where sensory experiences ‘*would annoy me a lot more and really get to me*’ (Ella_ch_). Even the anticipation of the stimulus could feel unbearable, as Alex_ad_ noted:

*‘[. . .] there just seemed to be like loads and loads of people on the streets. Whenever I left [. . .] to do something [. . .] even though no one was barging into me, I just couldn’t cope with it at all*’.

Henry_ad_ elaborated:

*‘It was like an affront to all my senses. It was like I was being in a boxing ring and I was being batted by somebody much, much bigger than me, you know? [. . .] I’ve become so sensitive to sound to the point where it makes me very angry’*.

### Theme 3. You crave solitude and sensory rest

As a response to the exhaustion, increasing loss of functions and the external world feeling more acute, unpredictable and painful, there was a, sometimes desperate, urge for solitude and sensory rest. Ashley_ch_ urgently wanted to hide in a ‘*dark cupboard*’, while Henry_ad_ had a fantasy of buying a ‘*cave to live in*’ to have somewhere to ‘*get away from the human race for several days a week*’. We understood this need for solitude and sensory rest to be experienced by participants regardless of when they were diagnosed as autistic.

We interpreted the participants’ sensory and social experiences to be intimately linked, in that sensory overload exhausted the participants, which left them without resources to maintain their usual social interactions. Several participants described finding others incomprehensible and increasingly intolerable, both before and during burnout. The lack of bandwidth to stay around others and overwhelming sensory input sometimes led to being ‘*agitated*’ (Esther_ch_), ‘*less patient*’ with (Diana_ad_) and ‘*intolerant*’ of (Henry_ad_) others, with participants preferring to spend time alone. During burnout, a few participants experienced a fear that they could snap if they did not get out of an overwhelming situation, with a few later-diagnosed participants sharing experiences where they did indeed lose control, abruptly leaving situations or throwing furniture.

### Theme 4. Making the world more bearable by using substances

Although we did not interpret using substances as inherent to the experience of autistic burnout nor as a coping mechanism solely reserved for autistic burnout, we understood it as coming in conjunction with burnout for some later-diagnosed participants. Used substances included food (‘*I probably comfort eat*’), drugs (‘*I did self-medicate a lot in my 20s [. . .] mostly marijuana*’), alcohol and cigarettes (‘*alcohol and the other one was tobacco*’).

Using substances seemed to have the effect of making experiences associated with burnout – its contributors and/or consequences – more manageable. For example, participants_ad_ spoke about how alcohol helped to create a ‘*safe space*’ from the social, sensory and emotional overwhelm tied to burnout:

*It calms me. And [. . .] it sort of puts me into this little state of equilibrium, where I have a couple of drinks and it’s just: ‘Okay. I can cope with the world now. I can cope with social interactions’*.

All participants who mentioned the use of substances related to their use as, for example, ‘*dangerous*’, ‘*self-harm*’ and a ‘*bad thing*’. Despite the communicated danger to their health, a few participants shared that trying to curb their use could make them feel unable to cope, even contributing to deeper burnout. One participant_ad_ stated:

*‘[. . .] at the very beginning of [the two-year burnout], I gave up drinking. [. . .] And what happened was, I got worse. Because the drinking helped me to cope*’.

### Theme 5. Not knowing why this is happening to you takes a (sometimes dangerous) toll

A feeling that overlaid these experiences of burnout, and seemed to amplify them, was confusion. Confusion could be experienced by participants regardless of diagnosis age, especially at the first occurrence of burnout when the person had never before encountered it. Regardless of diagnosis age, for several participants, it was also hard to listen to, understand and translate the needs of the mind and body. For participants diagnosed in adulthood, this disconnect from their mind and body seemed more extensive. It consisted of *living life without a necessary frame of reference* and, for a few, there was *a feeling there was no way out* from burnout.

### Living life not knowing you are neurodivergent means living life without a necessary frame of reference

For the participants diagnosed in adulthood, the difficulty in interpreting their emotional, psychological and physical experiences, including those relating to autistic burnout, was further associated with not knowing that they were neurodivergent. All later-diagnosed participants referenced this ignorance as creating an additional layer of distress during burnout.

We understood this distress to partly stem from having an unhelpful frame of reference for one’s needs in the context of the loud, frightening and incomprehensible world they lived in and experienced. Robert_ad_ described how, before understanding himself as autistic, he felt deep distress before, during and after burnout, due to his perceived inability to cope with the same experiences that everyone else was presumably going through:

Robert_ad_: ‘*I was thinking, like, I’ve [. . .] I’ve gone through this, and I’ve gone through that. But, like, I’m sure other people have done that [. . .] and coped [. . .] going through the same issues and thoughts and whatever that I am*’.

*DA: ‘Yeah. So [. . .] how can you not [. . .] cope?’*

*Robert_b_: ‘Yeah. Exactly*’.

Not knowing that they were neurodivergent was perceived by Helen_ad_ as leading to most of the distress associated with burnout, since not having the right frame of reference for herself meant she was also unaware of her true needs:

*‘I would say the not knowing created 80% of the problem. [. . .] Because once I know what the problem is, cognitively, I can go: great, right, what can I do to help myself? What can I do to solve it? What support can I get? What guidance? [. . .] I’m really good at problem-solving, but if I don’t know what the problem is, I can’t do it*’.

Prior to understanding themselves as autistic, several participants seemed to experience a felt sense of otherness during or following burnout, feeling ‘*self-loathing*’, seeing themselves as ‘*rubbish at life*’, ‘*weak [. . .] a failure*’ or ‘*weird*’. Coming to be recognised and understanding themselves as autistic meant not only an internal re-evaluation of one’s self-description and self-understanding but also the meeting of others who shared similar internal and external experiences. For Henry_ad_, finding out he was autistic and being surrounded by autistic others with a similar experience helped him create new meaning, one where he found more compassion for himself and where autistic burnout seemed an understandable reaction in a world ‘*not designed for most human beings*’:

‘*I just thought, actually, it’s OK to be in this state. [. . .] it’s almost like a sensible reaction to the too much pressure, the too much of everything that I’ve had to experience*’.

### You feel there is no way out

In the context of lacking the right support, understanding and compassion throughout their early lives, some later-diagnosed participants seemed to express a deep sense of entrapment and incomprehension in connection to their burnout specifically and their lives more broadly. One participant_ad_ explained:

*‘When your entire life has collapsed around your ears and [. . .] you’ve got no one to turn to, you’ve got nothing. . . It does feel like, you know, how do I get back from it? [. . .] At the time [. . .] when you haven’t got the energy to, kind of, do anything, it just seems like [. . .] you’re going to be stuck like that forever*’.

In connection to burnout and its consequences, multiple later-diagnosed participants shared instances of thinking about taking, or attempting to take, their own lives. This state seemed to occur for several reasons. For one participant_ad_, autistic burnout worsened their pre-existing depression, which came with ‘*suicidal ideation*’. For others, their incomprehension about their lives and selves before understanding themselves as autistic, and the losses due to autistic burnout, became too difficult to carry:

*‘I’ve probably thought about suicide across my whole life, because I’ve often felt like a failure, unloved, unreachable. [. . .] I felt, you know: I hate the fact that I couldn’t cope with things. [. . .] I’ve lost everything. [. . .] Why am I in this world? I can’t function in this world. It’s not designed for people like me*’.

## Discussion

In this qualitative study, we aimed to explore how autistic burnout felt for 20 autistic adults living in the United Kingdom, some diagnosed with autism in childhood and some in adulthood. Using RTA as our methodology, we generated five themes. We interpreted our participants’ autistic burnouts to be experienced as a powering down of their minds and bodies, consisting of feeling exhausted and unable to take part in day-to-day life. At the same time, some participants experienced an overactivation of the mind and body, which consisted of greater sensory sensitivity, restlessness and increased anxiety, frustration and anger. Some participants diagnosed in adulthood coped with these experiences through using substances, especially alcohol. Particularly those diagnosed in adulthood further expressed confusion and distress associated with not knowing what was happening to them. For some participants, particularly those diagnosed later in life, this led to feelings of deep confusion and entrapment.

### How does ‘autistic burnout’ feel?

Consistent with experiences across existing research (e.g., Higgins et al., 2021), we understood our participants to feel exhaustion of varying durations and severities that could lead, for some, to feeling unable to take part in day-to-day life and an urgent need for social and sensory respite. During autistic burnout, regardless of age at autism diagnosis, several participants expressed a feeling of overactivation of their minds and bodies, particularly in the form of stronger feelings of anxiety, anger and ‘meltdowns’ as a response to overwhelm associated with social and sensory stimuli. This is again consistent with some qualitative studies that have reported on both child and adult participants experiencing increased feelings of anger and meltdowns during (the run up to) exhaustion and autistic burnout (Keville et al., 2021; Mantzalas, Richdale, Adikari, et al., 2022; Phung et al., 2021).

Yet some of our participants diagnosed in childhood were interpreted to not strongly differentiate between their felt perception of ‘autistic burnout’ and ‘meltdown’. Both meltdowns and autistic burnout are associated with a felt internal overwhelm and loss of specific abilities, such as within memory and thinking (Lewis & Stevens, 2023). Meltdowns, however, are usually understood as briefer, and more externalising, whereby a person may communicate their distress more visibly, for example, through behaviour perceived as aggressive (Lewis & Stevens, 2023). Meltdowns are also understood to increase in frequency as burnout approaches (Phung et al., 2021), which may also be why some participants found it difficult to distinguish between the two experiences. It is, of course, possible that some of our participants were describing different experiential phenomena. However, the delineation between meltdowns and burnouts may not always be so straightforward, as captured by Phung et al. (2021), in whose study the interviewed autistic children and young people did not always communicate a clear boundary between which embodied experience was which. Perhaps for this reason, Phung et al. (2021) referred to the range of these overlapping experiences within a single acronym: ‘Burnout, Inertia, Meltdown and Shutdown (BIMS)’.

Not feeling able to take part in day-to-day activities (even when pushing through) was a pronounced feeling for many of our participants, especially those diagnosed with autism in adulthood. A few participants referred to ‘executive functioning’ explicitly. Executive functioning generally refers to a range of neurocognitive processes, such as inhibition and working memory (Demetriou et al., 2019). It is important to note there is a great variation in conceptualisation and operationalisation of executive function (for a review, see Baggetta & Alexander, 2016). In the context of autistic lives, burnout has been suggested to increase executive functioning difficulties (Arnold et al., 2023a); it has even been conceptualised as an ‘executive function and related trait’ in Ratto et al.’s (2023, p. 99) tool for the self-assessment of autistic traits. In extant general population literature, exhaustion, fatigue and burnout have been found to negatively impact executive function, cognitive processes and emotion regulation (Gavelin et al., 2021; Grillon et al., 2015; Koutsimani et al., 2021; Renaud & Lacroix, 2023), especially when demands are high (Diestel et al., 2013). As autistic people are thought to generally experience higher levels of executive functioning difficulties (e.g., Kofler et al., 2024; although this has been recently questioned – see Jertberg et al., 2025), the relationship between executive functioning and autistic burnout needs to be further explored. For example, does exhaustion related to burnout drive some of the executive functioning differences?

A key difference between our childhood- and adulthood-diagnosed participants was the seeming duration of burnout, with an apparently greater chronicity reported by those diagnosed with autism in adulthood. A partial explanation for this difference could be that the adult-diagnosed participants were generally currently older than those diagnosed in childhood. However, it is important to note that some of those diagnosed in later life did report difficulties, including with burnout, since childhood. Another partial explanation could be a differing perception of both autistic burnout and autism between the two groups. Although not all, those diagnosed in childhood seemed to generally express more confidence in their abilities to cope with burnout, even if they found it distressing. Rather than this being understood as solely an issue of individual resilience, it could be interpreted in the context of these earlier diagnosed participants having the right support earlier on, giving them the confidence and safety that allowed them to feel able to cope in turn. This study is very preliminary, however, and these interpretations are speculative.

Finally, we interpreted our participants’ felt need for solitude and sensory rest (whether shorter- or longer-term), which has been documented across studies in relation to autistic burnout recovery (e.g., Mantzalas, Richdale, Adikari, et al., 2021), to be adaptive. Solitude, when chosen, has been connected to wellbeing for people in the general (Weinstein et al., 2023) and autistic populations (Neville et al., 2024). It remains to be understood how withdrawing socially to recover from burnout impacts on autistic people long-term, and what supports and changes can be put in place to allow for a better balance between socialising and social respite.

### Experiences connected to, yet beyond, ‘autistic burnout’

Several of our participants seemed to use substances, primarily alcohol, to cope with the experiences that contributed to burnout (e.g., sensory overwhelm) or with the experiences that came with burnout (e.g., increased sensory sensitivity). It was not always easy to interpret if this coping mechanism extended beyond managing burnout, but, from some participants’ experiences, we understood this to be a more general coping mechanism. This is in line with a study that found neurodivergent people to, initially, find substance use helpful in coping with their difficulties (Kronenberg et al., 2014). Estimates of the prevalence of substance-use difficulties in autistic populations have not been consistent. It is possible prevalence may be higher for autistic adults, especially if they experience co-occurring neurodivergent conditions, for example, ADHD or mental health difficulties (Butwicka et al., 2017; Haasbroek & Morojele, 2022; Kaltenegger et al., 2021). Regardless, it is likely that routine substance-use support services may not be optimal for autistic people (Brosnan & Adams, 2022; Munday et al., 2025). Given the health consequences of substance use and dependence (GBD 2016 Alcohol and Drug Use Collaborators, 2018), understanding the prevalence and improving support acceptability for those on the autism spectrum who use substances and want, or need, to change their use is necessary.

Many of our – especially later-diagnosed – participants shared a sense of self-blame as a result of ability loss and difficulty with functioning at least partially connected to burnout. Some participants seemed to experience very severe distress, from which they perceived their only route of escape was to take their own lives. Suicide rates in the autistic population are significantly higher than in the general population (Brown et al., 2024). It has been suggested that the relationship between suicide and stigma can be reciprocal, in that belonging to a stigmatised minority group can increase the risk of suicide, and experiencing suicidality itself can be further marginalising (Carpiniello & Pinna, 2017; Oexle et al., 2018). It is therefore essential to further understand the relationship between autistic burnout and suicide, and how to support autistic people experiencing suicidality. Suicide prevention in the general population has often relied on risk prediction, an approach which has not been deemed particularly effective, with a recent call to take a more person-specific approach (NHS England, 2025). It will need to be further explored how these holistic and person-centred approaches can best be used and/or adapted for those on the spectrum.

Not understanding themselves as neurodivergent seemed to be related to a more distressing experience of autistic burnout for some of the participants. Recognising oneself as autistic can have positive impacts on some autistic people, supporting re-evaluation of one’s life (Kiehl et al., 2024), as well as having a deeper psychological benefit, specifically on one’s self-concept and sense of self-worth (Leedham et al., 2019; Stagg & Belcher, 2019). However, as with some of our participants, it is likely this identification with an autistic self may only have positive psychological benefits if a given person connects with an autistic identity (as not all who receive an autism diagnosis do) and views the autistic self positively (Nguyen et al., 2020; Oredipe et al., 2022; Pyszkowska et al., 2025; Tan, 2018). Hence, identification and diagnosis may not be enough; fostering a positive sense of self, such as through increased public acceptance of how individuals with needs that are not considered mainstream are generally understood and accepted, may be a necessary prerequisite to preventing autistic burnout specifically and improving autistic people’s quality of life more generally.

### Strengths and limitations of this study

We tried to conduct a community-engaged study. Albeit informally, we involved autistic people during the creation of the interview schedule and sought feedback from our participants on our initial findings, hoping to extend the collaborative approach to the analysis, with the lead researcher reflexively utilising this feedback. Furthermore, we hope that having an ‘insider’ perspective enriched data analysis through creating interpretations and generating patterns based on personal and professional experience that an outsider researcher may not necessarily have at their disposal (Greene, 2014). Nevertheless, being an insider researcher has its pitfalls, such as interpreting data through preconceived ideas based on one’s own experience (Jacobson & Mustafa, 2019). Hence, reflexive practices, such as keeping a reflexive diary and memos and having open discussions with team members, as with any RTA process, hopefully mitigated insider research pitfalls while drawing out its positives.

We worked to recruit participants from across diverse walks of life in terms of age, age at autism diagnosis, gender identity and ethnicity. Given that a significant amount of research related to autistic adults is conducted with late-diagnosed participants who identify as female, recruiting earlier diagnosed participants of male and female genders was one of this study’s strengths. Nevertheless, we did not manage to recruit as many participants belonging to gender and ethnic minorities as we had hoped, and we did not specifically interrogate participants’ socio-economic standing in relation to their burnout experiences, making these the study’s limitations. Although we did recruit participants diagnosed with autism in childhood (defined here as under the age of 18), few of these participants were diagnosed at a very young age – not necessarily early enough to provide clarity for their needs and difficulties as they were growing up. Indeed, several of the participants diagnosed in childhood indicated having experienced a sense of confusion and otherness similar to that experienced by participants diagnosed in adulthood. Furthermore, this study does not capture the experiences of those autistic adults whose cognitive or language abilities would have made it difficult to participate in this study. There is no research on severe fatigue and autistic burnout as experienced by autistic adults with a learning disability, and very little research conducted in languages other than English. Therefore, the present qualitative findings may not reflect the experiences of these populations.

Overall, the sample size was arguably on the smaller side, although there is no strict prescription for sample sizes in qualitative interview studies, with researchers needing to weigh the depth and detail of their data, with their research question(s) (Malterud et al., 2015). We judged that for the purposes of this study, with a strong quality of dialogue, combined with our aim, 20 participants were sufficient. The aim of qualitative studies is argued to be transferability, with readers making judgements on whether the presented findings resonate in similar contexts (Nowell et al., 2017) – as opposed to statistical generalisability and representativeness. Nevertheless, future quantitative studies could take up the relationship between age at autism diagnosis and ‘autistic burnout’ in larger samples to statistically explore differences in burnout experiences, for example, duration, between early versus late-diagnosed autistic people.

As noted in section ‘Findings’, the participants in the childhood-diagnosed group were, on the whole, younger than the adult-diagnosed participants. This means that some of the differences in the burnout experiences may have been due to the later-diagnosed (and older) participants having more negative life experiences to date, as well as experiences related to biological ageing itself. Nevertheless, in terms of the first point, these participants also described retrospective experiences that suggested that their experiences of burnout begun early in life. There may also have been other differences between the two groups, as well as factors impacting on both the timing of diagnosis and burnout experiences, that we did not take into account, and that could, at least partially, explain the differences in burnout we interpreted. For example, in the childhood-diagnosed group, more of the participants identified with a minoritised ethnicity. Although we tried to be sensitive to experiences of gender, age, ethnicity and culture when analysing the interviews, it is possible that meaningful differences were not captured in the interviews or that they were not part of our interpretation.

Finally, this study focused solely on burnout as experienced by a specific group of autistic adults, many of whom also identified with broader neurodivergence, particularly ADHD. It is important to note that burnout has been experienced by disabled people more generally (Wolbring & Lillywhite, 2023). To aid the conceptualisation of severe exhaustion and burnout within, and without, the neurodivergent population, future studies should explore lived experiences of people across the full range of neurodiversity.

## Conclusion

We aimed to explore how autistic burnout felt for earlier and later-diagnosed participants. We interpreted autistic burnout to be experienced as a powering down of the mind and body, as well as its overactivation, that left participants in need of solitude and sensory rest. For some participants, particularly those diagnosed with autism later in life, the experience was severe and chronic, with some coping through the use of substances, particularly alcohol. Not knowing they were neurodivergent left some participants without a necessary frame of reference to understand and address their needs, while understanding themselves as neurodivergent – and accepting themselves more – alleviated this confusion. Future studies should further explore the experiences of burnout across neuro/diverse populations to work towards supporting burnout recovery and aiding prevention.

## Supplemental Material

sj-docx-1-aut-10.1177_13623613261422117 – Supplemental material for How does ‘autistic burnout’ feel? A qualitative study exploring experiences of earlier and later-diagnosed autistic adults Supplemental material, sj-docx-1-aut-10.1177_13623613261422117 for How does ‘autistic burnout’ feel? A qualitative study exploring experiences of earlier and later-diagnosed autistic adults by Dorota Ali, Will Mandy and Francesca Happé in Autism
