# Supplementary material for: How does ‘autistic burnout’ feel? A qualitative study exploring experiences of earlier and later-diagnosed autistic adults
Source: Autism. 2026 Feb 28;30(4):1014–27. doi: 10.1177/13623613261422117 (PMC13005893; doi:10.1177/13623613261422117)
Supplement: sj-docx-1-aut-10.1177_13623613261422117 – Supplemental material for How does ‘autistic burnout’ feel? A qualitative study exploring experiences of earlier and later-diagnosed autistic adults [file sj-docx-1-aut-10.1177_13623613261422117.docx]

**Supplementary Materials**

**Interview Guide Shared with the Participants^[[1]](#footnote-1)^**

**Interview Themes (Autistic Burnout Study)**

***Opening questions.***

What does ‘autistic burnout’ mean to you?

Approx. how old were you when you first experienced autistic burnout?

How many times do you think you have experienced autistic burnout over your lifetime?

How long would you say autistic burnout lasted on average for you?

**Build-up to Autistic Burnout**

***Triggers and causes.***

What do you think has contributed to your latest experience of autistic burnout?

***Experience in the body.***

What signs did you notice in your body during the built-up to autistic burnout?

***Emotional experience.***

What did you notice about your emotions during the built-up to autistic burnout?

***Coping strategies.***

Were there any coping strategies you used, in order to try to prevent autistic burnout (if you noticed that you were heading in that direction)?

***Self and others.***

Tell me a little bit about how you felt about yourself during the build-up to developing autistic burnout.

**During Autistic Burnout**

***Nature of autistic burnout.***

Tell me what it was like experiencing autistic burnout.

***Time.***

How long did the recent/ has the current period of burnout last for you (so far)? Is this similar to your previous experiences?

***Experience in the body.***

What signs did you notice in your body during autistic burnout?

***Emotional experience.***

What did you notice about your emotions during autistic burnout?

***Self and others.***

How has autistic burnout affected your day-to-day life?

Has autistic burnout affected how you see yourself and what you feel about yourself and, if so, how?

How do you think being in autistic burnout affected your relationship with the people in your life?

***Treatment.***

Did you receive any treatment for autistic burnout (or a condition that you believe you were misdiagnosed with)? Could you tell me a little bit more about this?

***Coping strategies.***

Could you tell me a little bit about any coping strategies that helped you manage day-to-day when you were in the middle of autistic burnout?

***Related conditions.***

Are there any other experiences that you feel are similar to your experiences of autistic burnout?

**After Autistic Burnout**

***Nature of recovery.***

Tell me about your experience of coming out of a period of autistic burnout.

***Experience in the body.***

What signs did you notice in your body that meant you were recovering from autistic burnout?

***Emotional experience.***

What did you notice about your emotions during/after autistic burnout recovery?

***Recovery experiences.***

Are there any things in the long-term that help you maintain your recovery from autistic burnout (if this is applicable for you)?

**Closing question/s**

What are the key things you want others (loved ones, healthcare providers, society) to know about autistic burnout?

What would you say to another autistic person experiencing autistic burnout?

1. Please note this Interview Schedule is also submitted with [reference removed for blinded review], as both studies were based on the same interviews, and on the same participants. [↑](#footnote-ref-1)
